# The Role of α-Synuclein in Etiology of Neurodegenerative Diseases

**DOI:** 10.3390/ijms25179197

**Published:** 2024-08-24

**Authors:** Daria Krawczuk, Magdalena Groblewska, Jan Mroczko, Izabela Winkel, Barbara Mroczko

**Affiliations:** 1 Department of Neurodegeneration Diagnostics, Medical University of Białystok, 15-089 Białystok, Poland; daria.krawczuk@sd.umb.edu.pl (D.K.); mjanek2003@gmail.com (J.M.); 2 Department of Biochemical Diagnostics, University Hospital in Białystok, 15-269 Białystok, Poland; magdalena.groblewska@uskwb.pl; 3 Dementia Disorders Centre, Medical University of Wroclaw, 50-425 Ścinawa, Poland; i.winkel@me.com

**Keywords:** α-synuclein, neurodegeneration, Parkinson’s disease, dementia with Lewy bodies, multiple system atrophy, synucleinopathies

## Abstract

A presynaptic protein called α-synuclein plays a crucial role in synaptic function and neurotransmitter release. However, its misfolding and aggregation have been implicated in a variety of neurodegenerative diseases, particularly Parkinson’s disease, dementia with Lewy bodies, and multiple system atrophy. Emerging evidence suggests that α-synuclein interacts with various cellular pathways, including mitochondrial dysfunction, oxidative stress, and neuroinflammation, which contributes to neuronal cell death. Moreover, α-synuclein has been involved in the propagation of neurodegenerative processes through prion-like mechanisms, where misfolded proteins induce similar conformational changes in neighboring neurons. Understanding the multifaced roles of α-synuclein in neurodegeneration not only aids in acquiring more knowledge about the pathophysiology of these diseases but also highlights potential biomarkers and therapeutic targets for intervention in alpha-synucleinopathies. In this review, we provide a summary of the mechanisms by which α-synuclein contributes to neurodegenerative processes, focusing on its misfolding, oligomerization, and the formation of insoluble fibrils that form characteristic Lewy bodies. Furthermore, we compare the potential value of α-synuclein species in diagnosing and differentiating selected neurodegenerative diseases.

## 1. Neurodegenerative Diseases

Neurodegenerative diseases (NDs) form a spectrum of chronic, progressive diseases within the nervous system, the common feature of which is degeneration of the structure and function of the central nervous system (CNS) or peripheral nervous system. Many neurodegenerative conditions progress to dementia, which is predicted to affect approximately 150 million people globally in 2050 with an economic burden of USD 10 trillion. Neurodegeneration is an irreversible process characterized by progressive loss of neurons in different areas of the brain [1]. NDs are often associated with neuroinflammation and activation of microglia, the primary immune cells in the CNS, which results in disruption of the physiological homeostasis of nerve cells [2]. In normal conditions, microglia are resident cells of the brain that regulate brain development, maintenance of neuronal networks, and injury repair. Activated microglia are part of the self-defense of CNS and secrete many soluble factors, such as cytokines, chemoattractants, and neurotropic factors that contribute to various aspects of immune responses and tissue repair in the CNS. They destroy pathogens, remove damaged cells, eliminate toxic substances, prevent the spread of infections and injuries, release neurotrophic factors, and promote tissue repair and regrowth. Alterations in functionality of microglia are implicated not only in brain development and aging but also in neurodegeneration. In addition, ongoing neuroinflammation in NDs is associated with oxidative stress, as well as the formation of toxic forms of oxygen and free radicals [3].

Accumulation of misfolded proteins may occur in various tissues and cells. Deposition of these proteins can also result in a disturbed function of organs. For example, their accumulation in neurons leads to the formation of amyloid senile plaques, neurofibrillary tangles (NFTs), and Pick bodies consisting of tau protein [4]. These misfolded proteins may even exert a toxic influence on cells and tissues and can trigger the further misfolding of other proteins and their accumulation into other aggregates or oligomers, as is observed in prion proteins [5]. The phenomenon of protein aggregation and the formation of their deposits has become the subject of rapidly increasing research activities.

Specifically, α-synuclein (α-Syn) has gathered significant attention, particularly in the context of Parkinson’s disease (PD) and other synucleinopathies. α-Syn is increasingly recognized as a biomarker in the diagnosis and monitoring of neurodegenerative diseases, as its abnormal aggregation in the form of Lewy bodies is a hallmark of these conditions. A growing body of literature points to the fact that CSF levels of different α-Syn species are significantly altered in neurodegenerative diseases. Moreover, the presence of specific forms of this protein may help in the differentiation between various types of dementia [6,7]. Therefore, the aim of the current study was to review the role of α-Syn in etiology of selected neurodegenerative diseases. 

## 2. α-Synuclein Characterization

α-Syn is a small, 14-kDa protein translated from five exons of the SNCA gene. It exists in a balance between the soluble and membrane-bound form in neurons. The soluble form of α-Syn is natively unstructured whereas the membrane-bound state adopts α-helix. Aggregated α-Syn is the major constituent of the intraneuronal inclusions, i.e., Lewy bodies (LBs) and Lewy neurites (LNs), which represents the major pathological hallmark of Parkinson’s disease and dementia with Lewy bodies (DLB). Moreover, α-Syn is likely the major component of glial and neuronal inclusions in multiple system atrophy (MSA) [8].

### 2.1. Structure of α-Syn 

**Figure 1:**
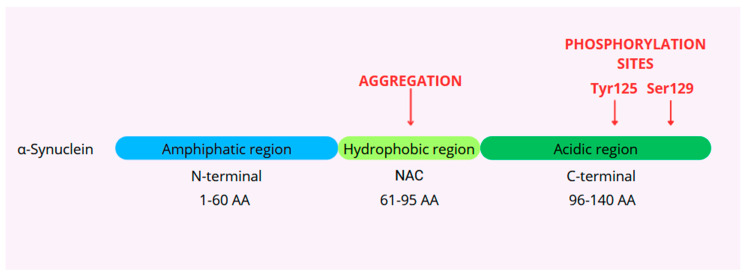
Structure of α-synuclein.

There are three main regions in the primary structure of α-Syn, which are characterized by different molecular and biological properties [9]. The domain structure of α-Syn consists of an amphipathic N-terminal region (1–60), non-amyloid component (NAC) (61–95), and C-terminal region (96–140). The N-terminal region of α-Syn is characterized by the presence of seven highly conserved 11 amino-acid repeat sequences (XKTKEGVXXXX) that form an amphipathic α-helix, which enables the protein to bind with membranes. This region includes the sites of three familial PD mutations. The hydrophobic NAC region is amyloidogenic and responsible for protein aggregation due to its ability to adopt β-sheet structures. It represents a second major intrinsic constituent of Alzheimer’s plaques [10]. The C-terminal domain is negatively charged and is involved in Ca^2+^ binding and chaperone-like activity. This domain contains most of the post-translation modification sites and mediates the interaction of α-Syn with other proteins, ligands, and metal ions [8]. It is suggested that the C-terminal domain interacts with the N-terminal or NAC to stabilize the native unfolded structure preventing fibrillation and forming monomeric structures. It also binds to calmodulin, calcium, and synaptic vesicles [11]. Figure 1 shows the structure of α-Syn.

### 2.2. Expression and Localization of α-Syn

Expression of α-Syn is apparent in the cerebral cortex, cerebellum, striatum, thalamus, hippocampus, and olfactory bulb [11] Apart from CNS localization, α-Syn is also present in various non-neuronal tissues, such as skeletal muscle and myocardium, kidney, liver, spleen, lung, bladder, and skin fibroblasts [12]. α-Syn is synthesized in the cell body, transported along the axon, and accumulated in the presynaptic part of nerve endings around synaptic vesicles [13]. It has been demonstrated that the axonal transport rate of α-Syn is significantly slowed down in the course of aging, which may facilitate the accumulation and aggregation of this protein in axons [14]. It has been also revealed that α-Syn has the ability to cross the blood–brain barrier (BBB) in two-way directions: brain-to-blood and blood-to-brain [15]. Thus, the transport of α-Syn within extracellular vesicles from erythrocytes across BBB may be a possible mechanism of the initiation and progression of PD [16]. 

### 2.3. Post-Translational Modifications of α-Syn

After translation, α-Syn is subjected to extensive post-translational modifications (PTMs), which may influence its toxicity and aggregation. PTMs of α-Syn include phosphorylation, ubiquitination, O-glycosylation, nitration, and truncation.

#### 2.3.1. Phosphorylation

It is estimated that under physiological conditions less than 5% of the soluble, monomeric α-Syn is phosphorylated. Conversely, approximately 90% of α-Syn is phosphorylated in LBs, suggesting a close relationship between phosphorylation at serine 129 (S129P) and α-Syn aggregation in PD and other synucleinopathies [17]. Phosphorylation of α-Syn may contribute to the regulation of the physiological functions of this protein such as membrane binding, oligomerization, fibril formation, and neurotoxicity, playing a critical role in PD and other synucleinopathies. It has been shown that phosphorylation in major sites, i.e., serine 87 (S87), and S129, inhibits α-Syn aggregation [18]. The main kinases that mediate the α-Syn S129 phosphorylation are G-protein-coupled receptor kinases (GRKs), casein kinase II (CK-II), polo-like kinases (PLK), and leucine-rich repeat kinase 2 (LRRK2) [19]. Zhou et al. revealed that whereas soluble S129P was detected in controls with higher levels in putamen compared with the frontal cortex, insoluble α-Syn occurred in PD with a significant increase in soluble and lipid-associated S129P and a decrease in soluble frontal α-Syn over the disease course. These findings suggest that soluble non-phosphorylated α-Syn decreases over the course of PD, becoming increasingly phosphorylated and insoluble [20].

#### 2.3.2. Ubiquitination 

Ubiquitin (UB) is a low-molecular protein with a molecular weight of 8.6 kDa, which consists of 76 amino acid (AA) residues. Its name comes from its ubiquitous presence in all eukaryotic cells. The addition of UB to a protein, called ubiquitylation, may alter the functions of the substrate in many ways, e.g., marking them for degradation, influencing their activity, and changing their cellular location [21]. Ubiquitylation is a reversible post-translational modification of various cellular proteins, including α-Syn [22]. It has been shown that the Lewy bodies are immunoreactive for both α-Syn and UB proteins, which may confirm the involvement of ubiquitination in the pathological properties of α-Syn [23]. Within Lewy bodies, phosphorylated α-Syn species may be mono-, di-, or tri-ubiquitinated [24], which involves activity of various enzymes, ubiquitin–protein ligases, such as C-terminal U-box domain of co-chaperone Hsp70-interacting protein (CHIP), seven in absentia homolog (SIAH) and neuronal precursor cell-expressed, developmentally down-regulated gene 4 (Nedd4) [25]. The result of the ubiquitination of α-Syn by CHIP is the degradation by two discrete mechanisms: the proteasomal and endosomal–lysosomal pathways [26]. Moreover, ubiquitination of α-Syn in vitro by SIAH promotes the formation of higher molecular weight forms of this protein and enhances its aggregation [27].

#### 2.3.3. O-Glycosylation

Another type of PTM of α-Syn, closely related to ubiquitination, is the O-glycosylation process. In a healthy brain, O-glycosylated α-Syn is present in very low concentrations, at the limit of detection. O-glycosylated α-Syn undergoes ubiquitination with the participation of the Parkin protein, which acts as a ubiquitin–protein ligase, and is then degraded by the proteasomal pathway [28]. In a hereditary variant of PD, the process of α-Syn ubiquitination is disturbed, which is associated with a mutation in the gene encoding Parkin, leading to impaired degradation of α-Syn and the accumulation of significant amounts of its O-glycosylated form in neurons. Since appropriate degradation of α-Syn is an important factor in preventing the formation of toxic forms of this protein, the disturbed degradation of O-glycosylated α-Syn may be a key factor leading to the death of dopaminergic neurons [29].

#### 2.3.4. Nitration

Among the various PTMs of α-Syn, nitrative modifications belong to the least favorable and most undesirable changes in this protein. A wide range of α-Syn nitration modifications can be detected in Lewy bodies, not only in PD patients, but also in other neurodegenerative diseases, such as DLB, MSA, and the Lewy body variant of Alzheimer’s disease. It has been shown that over 1/3 of the proteins deposited in Lewy bodies, are post-translationally modified (S-nitrosylated) by reactive nitrogen species (RNS) [30]. There are four tyrosine residues within the α-Syn molecule (Y39, Y125, Y133, and Y136), located in the N-terminal region and the C-terminal region, all of them susceptible to nitration [31]. In this modification, the -NO_2_ nitro group replaces a hydrogen atom in the 3′ position of the tyrosine phenolic ring to form 3-nitrotyrosine [32]. It has been revealed that depending on which tyrosine residue is nitrated, modified molecules of α-Syn exhibit distinct aggregation properties [31]. It has been also demonstrated that nitration of Y-39 is the modification that especially accelerates the oligomerization of α-Syn. Moreover, a mutation in this tyrosine residue leads to high levels of fibrilization in a cellular oxidative model of PD [33]. Nitration of α-Syn occurs in the major filamentous form of this protein and in the insoluble fractions of affected regions of the brain in patients with synucleinopathies, including PD [34]. Selective nitration of α-Syn is closely linked with oxidative damage in synucleinopathy lesions, leading to neurodegeneration, indicating that oxidative injury is also implicated in the pathogenesis of PD [35]. Moreover, monomeric and dimeric forms of nitrated α-Syn can accelerate the formation of fibrils and influence the fibrillation of non-modified α-Syn [36]. Nitration of α-Syn may also influence its membrane-binding capacity, which is mediated by the N-terminal region AA 1–95 of α-Syn. Therefore, nitration of tyrosine residue Y39, which is located within the N-terminus, may interfere with the binding of α-Syn to membrane lipids through the effect of repelling the same electrical charges in this area of the molecule [37].

#### 2.3.5. Truncation

It is estimated that about 15–20% α-Syn in LBs is truncated. This shortened form of α-Syn is found in association with α-Syn aggregates and may play a precipitating role and accelerate the aggregation of the full-length protein as well as high molecular weight α-Syn species [38]. α-Syn is truncated at the C- and N-terminals, including the NAC region due to the incomplete degradation by proteasomes and lysosomes [39]. Enzymes that may play a role in the proteolytical degradation of α-Syn and the generation of its C-terminally truncated species include Cathepsin D, which seems to be the main lysosomal enzyme involved in the degradation of α-Syn [40], as well as matrix metalloproteinase 3 (MMP-3) [41], and neurosin [42]. Contrary to C-terminal truncations, cleavage of the N-terminal and NAC region does not form aggregation-prone conformers. Many studies have indicated that the truncation of the N-terminal region decreases the ability of α-synuclein to polymerize. Thus, whether the truncated species aggregate depends on the enzyme involved in proteolysis as well as on the region of α-Syn that is cleaved [43].

## 3. Biological Functions of α-Synuclein

α-Syn interacts with a variety of proteins and performs a number of functions in conjunction with these protein interactions [18]. Taking into consideration that α-Syn has mainly presynaptic expression, it has been suggested that α-Syn plays a regulatory role in the activity of synaptic endings [44]. In addition, α-Syn may be involved in the compartmentalization, storage, and recycling of neurotransmitters. It has been revealed that α-Syn impairs the release of various neurotransmitters, such as dopamine and other catecholamines [45]. Another function of α-Syn is the regulation of the kinetics of synaptic vesicle endocytosis [46]. Increased expression of this protein leads to the restriction of synaptic vesicle mobility and inhibition of their reclustering after endocytosis. This leads to attenuation of synaptic vesicle recycling and reduces neurotransmitter release [47]. α-Syn acts also as a molecular chaperone to assist the folding and unfolding of soluble *N*-ethylmaleimide-sensitive factor attachment protein receptor (SNARE) proteins [48]. Neurotransmitters are released repeatedly from presynaptic nerve terminals and often at high frequency. This requires continuous cycles of assembly and disassembly of SNARE complexes, which mediate synaptic vesicle release at the neuronal synapse, vesicle recycling, and synaptic integrity. It has been demonstrated that α-Syn directly binds to the SNARE protein synaptobrevin-2 and promotes the formation of the SNARE complex [49].

Another recognized function of α-Syn, related to its localization in neuronal nuclei, is the role of DNA binding protein and its involvement in processes of DNA repair. The form of DNA damage is double-strand breaks (DSBs), which occur when both DNA strands are disrupted, as a result of exposure to ionizing radiation, certain chemicals, or as a result of programmed formation. DSBs lead to mutations, loss of heterozygosity, and chromosome rearrangements, resulting in cell death or dysfunction. It has been revealed that nuclear α-Syn may be involved in the processes of DSB repair. Markers of the response to DSB damage were co-localized with α-Syn, forming isolated foci in mouse brains and in cultured human cells [50]. Reduced expression of α-Syn in human cells caused increased formation of DSBs after exposure to bleomycin, a known factor inducing DNA strand breaks, leading to a reduced ability to repair these DSBs. In addition, α-Syn deficient mice displayed a higher level of DSBs, and this problem could be rescued by the reintroduction of transgenic human α-Syn, which promoted a DSB repair pathway termed “non-homologous end joining” [50].

**Figure 2:**
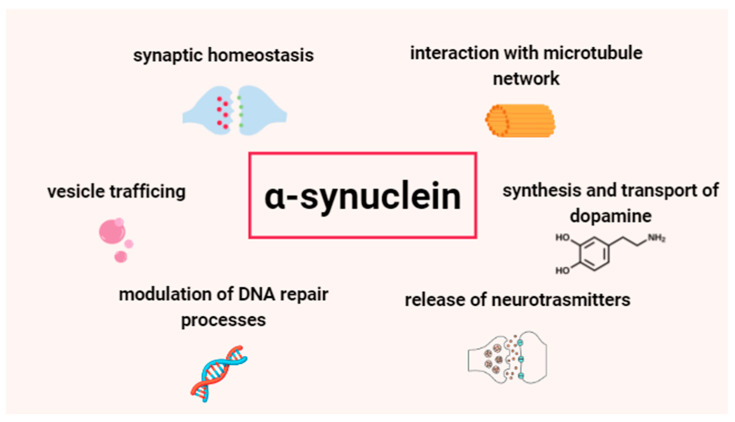
Biological functions of α-synuclein.

Apart from binding proteins, α-Syn has also ability to bind fatty acids. This protein occurs in high molecular weight complexes rich in lipids, especially fatty acids [51]. It has been shown that the interaction between monomeric α-Syn and fatty acids accelerated the formation of α-Syn assemblies, while the toxicity of these oligomers depended on the concentration of monomeric α-Syn [52]. Furthermore, α-Syn shows homology to the fatty acid-binding proteins (FABP), especially heart-type FABP (hFABP, FABP3), which associates with the aggregates of α-Syn. FABP3 is expressed by dopaminergic neurons and interacts with dopamine D2 receptors, which mediate α-Syn uptake [53]. Figure 2 shows the most important biological functions of α-synuclein.

## 4. α-Syn Misfolding, Aggregation, and Its Impact on Neurodegeneration

According to the “amyloid hypothesis”, which was initially developed for an explanation of Alzheimer’s disease (AD) pathogenesis, the aggregation of aberrant proteins into an ordered fibrillar structure may be the basis and cause of various neurodegenerative processes that finally lead to neuronal dysfunction and cell death [54]. However, the actual nature of the toxic species remains unknown.

### 4.1. Fibrillar Aggregates of α-Syn and Their Activity

**Figure 3:**
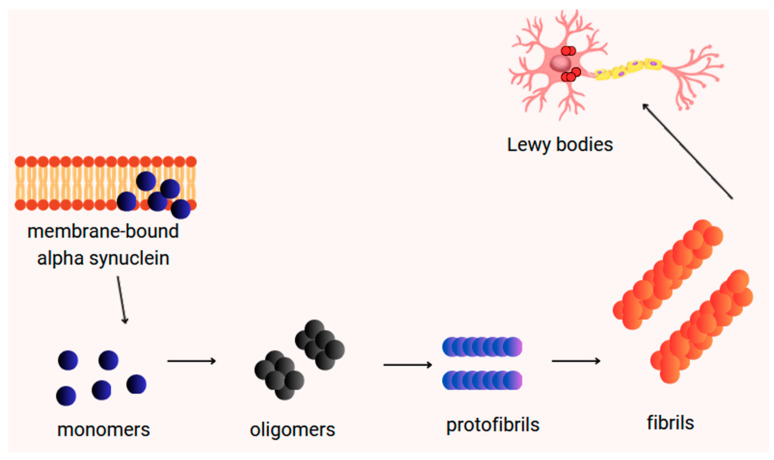
Formation of α-Syn oligomers and fibrils.

Abnormal fibrillation and subsequent aggregation are typical for α-Syn. As mentioned above, amyloid fibrils of α-Syn are the main components of Lewy bodies and Lewy neurites [55]. The fibrillar form is the most thermodynamically preferred state of α-Syn, mainly due to stabilizing interactions formed between monomers. The thermodynamic stability of α-Syn fibrils and their folding preference is dependent on the NAC region of the α-Syn molecule. However, there are different fibrillar types, formed at different rates. Because α-Syn fibrils develop from a variety of intermediate oligomeric species, they are also characterized by a diversity of forms [56]. α-Syn fibrils express certain features characteristic of typical amyloid proteins. In a study conducted in vitro, both fibrils generated from wild-type and those formed from mutant forms of α-Syn have been revealed to possess very similar features specific for amyloid β fibrils, such as predominantly unbranched morphology, antiparallel β-sheet structure, as well as similar dye-binding properties [57]. Moreover, α-Syn fibrils were relatively resistant to proteolysis, another property shared by fibrillar amyloid β (Aβ) and the disease-associated fibrillar form of the prion protein. These data suggest that PD, like AD, is a brain amyloid disease that, although, unlike AD, is characterized by endocytoplasmic deposits in Lewy bodies. Furthermore, apart from amyloid-like fibrils, small oligomeric forms of α-Syn were also observed, prior to the appearance of α-Syn fibrils. The formation of α-Syn oligomers, which may be responsible for neuronal death, rather than the α-Syn fibril itself, is another similarity to the Aβ protofibrils [58]. Figure 3 shows the formation of α-Syn oligomers and fibrils. 

Since Lewy bodies occur as cytoplasmic neuronal deposits containing α-Syn in a form that resembles fibrillar Aβ derived from AD amyloid plaques, the question was raised whether they could play a similar role in PD as amyloid aggregates in AD. It has been shown that the formation of fibrillar forms of α-Syn can be stimulated in vitro by heparin and other glycosaminoglycans (GAGs). Heparin not only increased the rate of α-Syn fibrillation but also the yield of fibrils in a concentration-dependent manner [59]. Moreover, in the presence of heparan sulfate and other electrically charged polymers, such as dextran sulfate, the formation of α-Syn fibrils was significantly stimulated. Interestingly, fluorescein labeling demonstrated that heparin was not only a catalyst for α-Syn fibrillation, but also integrated into the fibrils with a strong affinity [59]. Since there is some evidence that Lewy bodies may contain GAGs, these observations may be relevant in the context of the etiology of PD.

The exact molecular mechanisms by which fibrillar aggregates of α-Syn become toxic are poorly characterized. It has been shown that administration of α-Syn fibrils in vitro resulted in the activation of microglia cells and their inflammatory pathways, such as extracellular secretion of IL-1β [60]. Moreover, the spread of α-Syn fibril-induced inclusions in rats and mice correlates with neurodegeneration of dopaminergic neurons, thus suggesting that inclusion spread in the brain may be promoted by a loss of neurons [61].

### 4.2. Oligomer Polymorphism and Toxicity

Generation of α-Syn fibrils is a multi-step, complex process. At the early stage of the polymerization, a whole range of different oligomers of α-Syn is formed. Since α-Syn is a highly dynamic protein, it can access a large number of different assembly states apart from the fibrillar form, i.e., di-, tri-, tetramers, and various oligomers, which additionally can interconvert one into another [62]. The oligomerization of α-Syn results in the synthesis of heterogenous oligomers ranging from small (~2–5-mers), medium (~5–15-mers), to large (~15–150-mers) species [63]. Depending on different conditions of formation, α-Syn oligomers may vary in molecule shape, content of β-sheet conformation, and stability [64]. Moreover, these oligomeric assemblies of α-Syn also express distinct secondary structures, implying variegated toxicity under disease conditions [65]. Additionally, some oligomers may occur as “on-pathway” intermediate forms or as “off-pathway” dead-end products in the process of forming mature fibrils [66]. The oligomers whose blockage results in an apparent prevention of fibril formation are described as “on-pathway” oligomers. On the contrary, off-pathway oligomers have the possibility of the eventual formation of fibrillar assemblies [67]. It seems that both on- and off-pathway oligomers may be implicated with neuronal toxicity.

Similarly, to the postulated role of Aβ oligomers in the development of AD [68], it is believed that α-Syn oligomers are the more harmful form in neuronal impairments and disease progression. It seems that they may provoke more detrimental damages in neuronal cells, and thereby exacerbate α-synucleinopathy, compared to mature fibrillar forms of α-Syn [69]. In addition, α-Syn oligomers manifest varying toxicity profiles dependent on the specific environments as well as the shapes and conformations the oligomers adopt. 

Both oligomeric and fibrillar species have been shown to be cytotoxic, although it is still under debate, which form of α-Syn is actually more noxious and detrimental for neurons. There are many attempts to bring together inconsistent observations regarding the cytotoxic roles of oligomers versus fibrils [70]. The relationship between oligomeric and fibrillar forms needs to be clarified. Interestingly, although the generation of oligomeric species under disease conditions is likely correlated to cytotoxicity and different cellular damages, α-Syn oligomers manifest varying toxicity profiles dependent on the specific environments as well as the shapes and conformations the oligomers adopt. As the disease progresses, these deleterious α-Syn aggregates undergo neuron-to-neuron propagation along the midbrain, progressively destroying dopamine-producing neurons in the substantia nigra [71].

#### 4.2.1. Amyloid Fibril Formation

The formation of diverse non-fibrillar aggregates can be identified with several standardized biophysical methods including electron microscope (EM), atomic force microscope (AFM), and fluorescence spectrometer at the onset of amyloidogenesis. Namely, the early stages of α-Syn fibrillation generate an array of distinct oligomeric species varying in size, shape, stability, and β-sheet content, particularly during the lag phase [64]. These oligomeric species can be either on-pathway intermediates or off-pathway dead-end products, however, most of them are considered unstable, transient intermediates arising in the path of forming mature fibrils [66]. Although the major difference between on- and off-pathway oligomers is distinguished by the eventual formation of fibrillar assemblies, some researchers only classify the oligomers whose blockage can lead to an apparent prevention of fibril formation as on-pathway oligomers [67].

#### 4.2.2. Amyloid Fibril Disaggregation

It has been demonstrated that mature α-Syn amyloid fibrils can disaggregate and release soluble polymorphic dimers and oligomers, which may provoke severe toxic outcomes to neurons in the vicinity. Interestingly, oligomers produced from short fibrils elicit significantly more deleterious effects in neurons when compared to species released from long fibrils. This is partly because the release of toxic oligomers occurs from the fibrillar ends, whereas shorter fibrils facilitate a faster release owing to their higher proportion of ends [72]. In addition to immediate functional impairments in the neurons, these oligomeric species can be internalized and contribute to the progressive diffusion of α-synucleinopathy through neuron-to-neuron transmission. Studies on denatured mature fibrils under supercooling conditions show annular and spherical oligomeric species, which reportedly exhibit similar toxicity levels to oligomers generated during amyloid formation [73].

#### 4.2.3. Binding to Lipid Membranes

The interactions between α-Syn monomers and the cell membrane are particularly critical during the initial stages of amyloid formation [74]. Upon binding to lipid membranes, α-Syn monomers oligomerize, primarily to dimers and trimers, as the cross-linking between α-Syn monomers acts to stabilize the conformations of membrane-bound α-Syn. Notably, the membrane-induced oligomerization of α-Syn may result in the formation of nucleation sites for subsequent aggregation and aggravated α-synucleinopathy. Some researchers suggest that a longer incubation of membrane-bound spherical oligomers results in structural conversion into membrane-bound annular oligomers, where such alteration may contribute to increased toxicity and accelerated disease progression [75].

#### 4.2.4. Relationships between Structure and Toxicity of α-Syn

Unlike mature amyloid fibrils, α-Syn oligomers are predominantly localized in the presynaptic terminals, where they exert harmful impacts on synapses and dendrites. Similar to their provocation of distinct fibrillation kinetics to mature amyloids, differently structured and shaped oligomers elicit distinctive toxic outcomes. The early onset of the fibrillation process typically produces small spherical oligomers, which further assemble into annular protofibrils or even mature fibrils in the presence of excess α-Syn monomers [64]. While the mechanisms underlying the toxicity of α-Syn oligomers primarily pertain to various forms of cell membrane perturbation, the interactions between spherical oligomers and the membrane are not particularly pronounced [76]. Therefore, compared to annular oligomers, spherical or globular oligomers are considered more stable and thus generally display less deleterious toxicity profiles [62]. In support of this notion, studies on brain tissue samples of multiple system atrophy patients manifesting α-synucleinopathy revealed that mild detergent treatment breaks apart the inclusions into 30–50 nm-sized annular oligomers. On the other hand, the same treatment on recombinant wild-type (WT) α-Syn results in the release of spherical oligomers [77]. The findings suggest that pathological conditions preferentially form annular oligomers with higher toxicity. However, it should be noted that, unlike annular oligomers, spherical oligomers can be internalized by neuronal cells and function as a seed for consequential nucleation and elongation [78]. 

Annular species have become the focal point for understanding the neuronal impairments induced by α-Syn oligomers under disease conditions. In addition to the studies with MSA patients’ brain tissue samples showing α-Syn inclusions are predominantly formed by annular species, several subsequent studies further validate the implications of annular oligomers in toxicity including the specific types of damages they provoke. In the past, the prevailing hypothesis around the toxicity of α-Syn oligomers proposed the embedment of annular species into lipid bilayers, which leads to the formation of pore-like protein channels [79,80]. However, further analyses have since corroborated that the intercalation of the oligomers between tightly packed lipid domains induces disintegration of the hydrophobic core where destabilized membrane permeability thus triggers an aberrant transport of molecules across the membrane [80]. Indeed, the updated notion with decreased lipid order is consistent with the observations of elevated lipid flip-flops induced by oligomers. Importantly, membrane destabilization can lead to dysregulation of intracellular calcium homeostasis. Several hypotheses consider atypically increased intracellular calcium levels as an important factor contributing to neurodegeneration [81].

## 5. α-Synuclein in Parkinson’s Disease

Parkinson’s disease, also known as idiopathic or primary parkinsonism, is the second most common neurodegenerative disorder after AD. Similarly to other NDs, the prevalence of PD increases with age, being at 1% for people over 60 years of age, and 3% for people over 80 years of age [82]. The histopathological features of PD include the loss of pigmented dopaminergic neurons and the presence of Lewy bodies. Other mechanisms such as neuroinflammation, mitochondrial dysfunction, and changes in the lysosomal and endosomal functions are involved in the pathogenesis of this disease [83].

PD is characterized by both motor and nonmotor features. Typical cardinal signs are resting tremor, muscular stiffness, bradykinesia, and posture instability. PD diagnosis is based on the existence of bradykinesia and at least one of the other cardinal signs. Typically, patients experience the motor features of PD after 50% to 80% of dopaminergic neurons have been lost, suggesting the involvement of compensatory mechanisms in the early stages of the disease [84]. Through its progressive degenerative effects on mobility and muscle control, PD has a substantial clinical influence on patients, their families, and caregivers. The risk factors for the disease include oxidative stress, the formation of free radicals, and a number of environmental toxins such as carbon dioxide, herbicides, and pesticides. Interestingly, an inverse relationship exists between cigarette smoking, caffeine intake, and the risk of developing PD [85]. The most common gene mutations associated with PD are the alpha-synuclein gene (SNCA), glucocerebrosidase gene (GBA), leucine-rich repeat kinase 2 (LRRK2), and PTEN-induced putative kinase 1 (PINK1) [83].

The diagnostic process of PD is quite challenging, particularly at the earliest stages, due to the fact that PD symptoms overlap with other diseases including essential tremor, PSP, or MSA. Additionally, it is difficult to differentiate PD from PD dementia, DLB, and Alzheimer’s disease. α-Syn is a promising candidate as its concentrations may reflect the accumulation and aggregation of misfolded α-Syn in Lewy bodies and Lewy neurites [86]. Table 1 presents the clinical application of α-Syn species in PD.

A plethora of studies have indicated decreased levels of total α-Syn in PD compared to healthy controls [87,88,89]. However, total α-Syn levels may exhibit underwhelming performance as a diagnostic biomarker due to the fact that such assays measure both physiologic and pathogenic form of α-synuclein (i.e., oligomers, fibrils or phosphorylated, ubiquitinated forms) and cannot discriminate between the two. Thus, many studies focus on the measurement of those pathogenic forms of the α-Syn, especially oligomers and S129P. Majbour et al. found significantly increased levels of both oligomeric and phosphorylated α-Syn among PD patients compared to healthy controls. Additionally, the oligomeric/total α-Syn ratio (o-/t-α-syn %) was also increased in PD group and improved the ability to discriminate PD from healthy controls [88]. Measurements of early aggregates of α-Syn, such as oligomers, may favor the early detection of PD since oligomerization of α-Syn precedes neuronal death in the disease. For example, although several studies have reported a significantly reduced level of total α-Syn in CSF of PD patients, the o-α-syn/t-α-syn ratio has been revealed to be the most sensitive and specific parameter to differentiate PD from controls [90].

The role of α-Syn in differential diagnosis is also under investigation. It has been shown that CSF α-Syn concentration was significantly lower in the postural instability gait difficulty (PIGD) type compared to the tremor dominant (TD) type of PD, suggesting a potential role of α-Syn in developing strategies for proper treatment, depending on PD subtype [87]. Moreover, it has been shown that total α-Syn levels were higher in PDD compared to PD indicating its possible role in monitoring disease progression. Although authors stated that a reduced number of samples for some of the α-Syn aggregation disorders impeded an accurate calculation of sensitivity and specificity, further analysis in a large cohort with established diagnosis will refine the accuracy of those findings [91]. Furthermore, many studies investigate whether α-Syn may be useful in differentiation between PD and AD. It has been revealed that α-Syn levels were significantly lower in PD compared to AD [92]. Additionally, total α-Syn concentration was significantly lower in the PDD group compared to AD. Contrarily, oligomeric α-Syn and o-α-Syn/α-Syn ratios were increased in PDD compared to AD. Both oligomeric and ratio could distinguish PDD patients from AD with good diagnostic accuracy [93].

Hall et al. investigated whether levels of α-synuclein change over time. They observed increased concentrations of α-Syn after 2 years in PD patients compared to controls. A more significant increase was observed among patients with long disease duration (>5 years). Moreover, those results correlated strongly with CSF tau, a marker of neurodegeneration. However, the authors admitted that the follow-up period was relatively short, especially given the fact that PD develops over many years. Thus, studies with longer follow-up periods are needed [94]. Similarly, a 2-year follow-up study also showed an increase in total α-Syn concentration. Additionally, authors measured other α-Syn species and revealed an increase in oligomeric α-Syn and a decrease in S129P. Furthermore, the o-α-Syn/α-Syn ratio was increased, whereas the S129P/α-Syn ratio was notably decreased. The o-α-Syn/α-Syn ratio was associated with UPRS motor scores [95]. In another study, Stewart et al. investigated the longitudinal role of S129P in the course of PD as well as its utility in preclinical cases. Authors observed a longitudinal increase in S129P and S129P/α-Syn ratio in recently diagnosed, untreated PD patients, especially those who progressed to requiring dopamine therapy. Moreover, high S129P concentrations reflected less severe symptoms at the earliest but not at later stages. Those findings could be explained by the fact that α-Syn phosphorylation increases as a compensatory mechanism. That is why increasing levels showed contradictory associations with less severe symptoms at early stages, however with disease progression S129P continues to increase and becomes associated with worse outcomes in patients with more advanced disease stages. Thus, the relationship between S129P and disease severity may alter with progression [96]. In another longitudinal study, it was revealed that higher baseline CSF α-Syn correlated with cognitive processing speed (AQT) worsening over a 2-year period. Moreover, increased baseline α-Syn levels were associated with worsening motor symptoms over 2 years. Thus, increased concentrations of α-Syn among PD patients may predict future cognitive decline and worsening in motor symptoms [97].

## 6. α-Synuclein in Dementia with Lewy Bodies

DLB is one of the most common forms of dementia after Alzheimer’s disease and has been shown to account for 3.8 percent of new dementia diagnoses, with prevalence estimates indicating that DLB accounts for 4.2 percent of dementia diagnoses in community settings and 7.5 percent of diagnoses in secondary care. Among people over the age of 75, DLB accounts for about 5% of all dementia cases [98]. Neuropathologically DLB shares similar hallmarks to PD, i.e., aggregation of α-synuclein species in the brain in the form of Lewy bodies and Lewy neurites. Accumulation of these proteins impairs the functioning of neurons, leading to the death of those neurons. DLB is frequently misdiagnosed with AD as cognitive decline is the first event. Further symptoms, including motor dysfunction, are not specific and may be found in other diseases, which makes differential diagnosis challenging. Patients with Lewy body dementia present with a variety of neuropsychiatric symptoms, including visual hallucinations, systematized delusions, apathy, aggression, anxiety, and depression [99]. The disease starts with the prodromal stage (pro-DLB) also known as mild cognitive impairment due to Lewy bodies. However, proper diagnosis at the earliest stage is difficult, especially when a patient presents with other comorbidities. Thus, specific biomarkers are needed to allow early diagnosis and differentiation from other neurodegenerative diseases [100]. Table 2 presents a clinical assessment of α-Syn species in DLB.

Total α-Syn levels decreased, whereas oligomeric α-Syn increased in DLB compared to controls. The reduction of total α-Syn level is likely due to its aggregation and sequestration in Lewy bodies. However, soluble oligomers could be more useful as it has been postulated that these early aggregates may play a more crucial role in the pathogenesis of α-synucleinopathies than late aggregates. Moreover, it has been revealed that oligomeric forms of α-Syn are more pathogenic and neurotoxic both in vitro and in vivo and are linked to synaptic and neuronal degeneration. One of the limitations of this study was the fact that authors used erythrocytes instead of hemoglobin to measure the contamination of red blood cells in CSF, which may have overestimated the actual erythrocyte count [101]. In a similar study, the oligomeric α-Syn/total α-Syn ratio was significantly higher in the DLB group compared to controls [93]. Llorens et al. investigated whether adding tau protein will improve the diagnostic accuracy of distinguishing DLB from controls. Indeed, results showed that the tau/α-synuclein ratio was higher in the DLB group and yielded a good clinical accuracy in discriminating controls from dementia with Lewy bodies cases compared to single α-Syn and tau levels [91]. Similar results were obtained by Parnetti et al. A significant increase in tau/α-Syn ratio was shown and it outperformed the diagnostic accuracy of α-Syn alone. Thus, there is an interplay between total tau and α-Syn which is supported by the fact that both of these proteins were found in synaptic-enriched fractions in synucleinopathies. Moreover, neurofibrillary tangles colocalize with Lewy bodies. Therefore, the reduction of total α-Syn and increase in the tau/α-Syn ratio could be due to the mutual interaction between α-Syn and tau in the brains of DLB patients. In conclusion, α-Syn alone lacks clinical value as a biomarker of α-synuclein-related disorders, but in combination with total tau, it may improve the diagnosis of dementia with Lewy bodies [103].

α-Syn is also investigated in terms of differential diagnosis with other neurodegenerative diseases. It has been demonstrated that α-Syn levels were significantly higher among DLB patients compared to PD and PDD patients. The authors established an optimal CSF α-Syn cut-off of 128.5 pg/mL for differentiation between PD and DLB as well as 78 pg/mL for differentiation between PDD and DLB. However, it should be noted that there was a relatively small sample size and no neuropathological confirmation of diagnosis [102]. Several studies evaluated the diagnostic value of CSF α-Syn in the differential diagnosis between DLB and AD [105,107]. Bousiges et al. found that total α-synuclein levels were significantly lower in the DLB group compared to the AD and prodromal AD group, suggesting that α-Syn levels are altered already in prodromal stages. A possible explanation of such a decrease is due to its aggregation but also patients with DLB tend to have less intensive neurodegeneration compared to AD patients. Those findings suggest that total α-Syn assay can aid in discriminating between DLB and AD patients and that the changes in α-Syn levels are implemented early [100]. In another study total α-Syn levels were the lowest in the DLB group compared to AD and PD but not FTD. Such a strong decrease in DLB patients may be explained by both synaptic loss and the sequestration of the α-Syn in the widespread cortical Lewy body pathology that takes place in this disease [103]. Contrarily, oligomeric α-Syn and o-α-Syn/α-Syn ratios were increased in DLB compared to AD. Both oligomeric and the ratio could distinguish DLB patients from AD [93]. When combining α-Syn with pTau181 levels, the discriminatory value between DLB and AD reached 85% sensitivity and 81% specificity. In comparison, the sensitivity reached when using clinical diagnostic criteria was 32.1%. Although Aβ1-42 is an established biomarker for AD, concomitant amyloid pathology in DLB limits the use of Aβ1-42 for the differential diagnosis between these diseases. Thus, CSF α-Syn in combination with p-Tau181 may add an additional value in distinguishing DLB and AD [104]. 

## 7. α-Synuclein in Multiple System Atrophy

Multiple system atrophy (MSA) is a rare, progressive neurodegenerative disease characterized by the combination of parkinsonism, cerebellar ataxia, and autonomic failure. Neuropathologically, it presents with glial cytoplasmic inclusions (GCI) consisting of α-synuclein in oligodendroglia as well as neuronal loss in striatonigral and olivopontocerebellar systems. Two major clinical subtypes include MSA-P with predominantly parkinsonian deficits and MSA-C with predominantly cerebellar deficits. Additionally, minimal change MSA (MC-MSA) is also characterized by neuronal loss although restricted to the substantia nigra and locus coeruleus [108]. The established criteria to diagnose MSA distinguish 4 subcategories according to clinical certainty: neuropathologically established MSA, clinically established MSA, clinically probable MSA, and possible prodromal MSA [109]. The diagnostic process of MSA is especially challenging due to overlapping with other neurodegenerative disorders such as DLB, PD, progressive supranuclear palsy (PSP), or spinocerebellar ataxia (SCA). Thus, patients are often misdiagnosed, especially during the earliest disease stages, which has a negative impact on their quality of life [110]. A recent study investigating MSA models with cognitive impairment demonstrated that α-Syn was first expressed in oligodendrocytes but then accumulated in the cytoplasm of excitatory hippocampal neurons and their presynaptic nerve terminals. These findings were associated with the onset of memory impairment. Accordingly, postmortem analysis of human MSA brain tissues showed that patients with memory impairment have more α-syn inclusions in excitatory hippocampal neurons along with α-syn oligomers than subjects without cognitive deficits [111]. Thus, more research focuses on the use of α-Syn species in MSA diagnosis as well as differentiation from other neurodegenerative diseases. Table 3 presents a clinical assessment of α-Syn in MSA.

Mean CSF total α-Syn levels were decreased in MSA patients compared to neurological controls. Such a decrease may be explained by a possible increase in the rate of α-Syn uptake from CSF into neurons and oligodendroglia, thus allowing intracellular inclusion formation and cell-to-cell propagation of synucleinopathy [112]. Similar results were obtained by other researchers [113,114,115,116]. As for the differential diagnosis total α-Syn levels in MSA patients were lower compared to PD and DLB [112]. Shi. et al. found that total α-Syn differentiates MSA from PD, as its concentration decreases more significantly in MSA. Moreover, the p-tau/tau ratio is increased in MSA compared to PD. Authors explain this with the fact that in MSA, there is more widespread or faster neurodegeneration compared to PD, as low α-Syn levels reflect a reduction of free α-Syn circulating in the CSF, likely due to aggregation. Thus, a combination of α-Syn and the p-tau/tau ratio may add a value in differential diagnosis between those two synucleinopathies. It should be noted, however, that the population of MSA subjects was relatively low [113]. Mondello et al. revealed that total α-Syn levels were notably lower in MSA compared to PD, PSP, and CBD [114]. Total α-Syn concentrations differentiated MSA patients from AD [115]. These findings are in line with another study [116]. Oligomeric and phosphorylated α-Syn concentrations were the highest in MSA compared to PD and DLB. Thus, raised levels of phosphorylated forms of α-Syn may provide a test not only for distinguishing MSA from healthy individuals but more importantly from other synucleinopathies [117]. Wang et al. also investigated the value of S129P in differential diagnosis of synucleinopathies and revealed that concentrations were lower in MSA than in PD. Although S129P levels were similar in MSA and PSP, the ratio of S129P to total α-Syn differentiated these two groups. Thus, assessing both CSF S129P and total α-Syn may enhance the accuracy of differential diagnosis among overlapping parkinsonian disorders, thereby improving the diagnostic classification, especially at the early stages of the disease [118].

## 8. α-Syn in Traumatic Brain Injury

Traumatic brain injury (TBI) refers to a brain injury caused by an outside force. It can result in memory and cognitive impairment, altered consciousness, depression, and other psychiatric disturbances, as well as physical impairments, which contribute to reduced quality of life. TBI is accompanied by a variety of processes including excitotoxicity, impaired mitochondrial function, axonal injury, and synaptic dysfunction [119]. The yearly incidence of TBI is estimated at 50 million cases worldwide. Thus, approximately half of the global population will have an episode of TBI in their life. The clinical severity of TBI is stratified according to post-resuscitation Glasgow Coma Scale (GCS) scores into mild (GCS 14–15), moderate (9–13), and severe (3–8) [120]. Repetitive head trauma is the main cause of developing chronic traumatic encephalopathy (CTE), although studies suggest that CTE may occur even after a single to moderate TBI episode. Pathologic features of CTE include cerebral atrophy involving the frontal and temporal lobes, thalamus and hypothalamus, and the mammillary bodies along with abnormal accumulation of hyperphosphorylated tau protein and α-Syn in neurons and astroglia. The first stage is usually asymptomatic, although headaches, disability to focus and depression may arise. During the second stage, patients usually develop short-term memory deficits, aggressive behavior, mood swings, and problems with organization. Within the third and fourth stage, the cognitive decline is evident [121].

It has been shown that PD and TBI brains show some similarities, such as neuronal degeneration, disruption of blood–brain barrier, infiltration and expansion of resident microglia into the affected areas as well as infiltration of phagocytic cells from the periphery [122]. Acosta et al. examined the link between PD-associated dopaminergic neuronal loss and chronic TBI. Immunohistochemical data revealed downregulation of tyrosine hydroxylase (TH)-positive cells accompanied by increased accumulation of α-Syn in the substantia nigra pars compacta (SNpc) of 60 post-TBI animals. Moreover, there was a significant effect of TBI on the density of α-Syn in the ipsilateral SNpc of rats subjected to chronic TBI [123]. Thus, a growing body of research tries to investigate whether α-Syn would have potential as a biomarker of TBI and CTE. Mondello et al. conducted a study examining CSF α-Syn levels of patients with severe TBI. CSF samples were obtained at admission and daily for up to 8 days after injury. It has been revealed that CSF α-Syn was significantly higher in TBI patients compared to controls. Moreover, patients who died had higher levels over 8 days of observation compared to those who survived 6 months postinjury. Those findings indicate that an increase in CSF α-Syn concentration among TBI patients reflects the widespread neurodegeneration. Thus, monitoring CSF α-Syn levels may predict outcomes after severe TBI with high diagnostic accuracy [124]. A similar study, although concerning infants and children, showed significantly elevated α-Syn levels compared to controls, with an early 5-fold increase on days 1–3, followed by a delayed >10-fold increase on days 4–6, compared to controls. Authors explain that initial increase may be due to a disruption of tissue with the release of intracellular components to extracellular space. The following rise on day 4 and after may reflect secondary mechanisms. Thus, brain injury may cause a release of α-Syn from injured neurons, and to a lesser extent from glial cells. Interestingly, patients treated with hypothermia exhibited lower levels of α-Syn compared to those treated with normothermia. It is possible that hypothermia reduces α-Syn transport and exocytosis, or its release may be blunted due to the neuroprotective effect of hypothermia against secondary injury mechanisms [125]. The abovementioned findings suggest that the temporal profile of CSF α-Syn may predict long-term outcomes after severe TBI. However, there are only a few studies in this area, therefore future research evaluating the clinical utility of α-Syn as a biomarker of secondary injury is necessary. Identification of α-Syn as a risk factor among the TBI and CTE populations may be crucial for therapeutic management or as a potential target of therapy [124].

## 9. Conclusions

The abnormal aggregation of α-Syn in the form of intra-neuronal Lewy bodies and Lewy neurites has proved to be the major pathologic hallmark of PD, DLB, and MSA. Moreover, α-Syn contributes to the fibrillization of amyloid-β and tau, two main proteins associated with Alzheimer’s disease, suggesting a central role of α-Syn toxicity in neurodegeneration. Due to the overlapping of symptoms among PD, DLB, and MSA, the definitive antemortem diagnosis of α-synucleinopathies is challenging. Thus, these pathologies may be misdiagnosed, leading to ineffective therapeutic interventions [126]. Although the exact physiological function of α-Syn remains unclear, its highly concentrated presence in presynaptic buttons of neurons suggests several functions related to neurotransmitter release and role in synaptic vesicle dynamics. Loss of physiological function is associated with the conversion of α-helix to pathological β-sheet form, which interferes with α-Syn ability to associate with membranes. Once converted to an insoluble, β-rich structure, it becomes highly prone to form protofibrils and fibrils [127]. Many factors are considered to be involved in the process of misfolding and aggregation of α-Syn, such as hereditary, micro-environmental (posttranslational modifications, lipid interactions, neuroinflammation, gut microbiota, oxidative stress), macro-environmental (toxins and metal ions exposure), and epigenetic factors. α-Syn begins its misfolding a long time before the onset of symptoms, therefore it is crucial to understand molecular mechanisms that promote such misfolding so that proper treatment could be implemented early enough [128]. A lot of research has been conducted in order to examine the diagnostic and differential value of α-Syn species in neurodegenerative diseases. Although the majority of studies focus on PD, a growing body of research examines other synucleinopathies, i.e., DLB and MSA. Assessing both total and oligomeric as well as phosphorylated α-Syn may enhance the accuracy of differential diagnosis between overlapping parkinsonian disorders, thereby improving diagnostic classification, especially at the early stages of the disease [118]. To conclude, α-Syn holds promise as a biomarker for neurodegenerative diseases, particularly synucleinopathies. Conducting longitudinal studies to investigate how total α-Syn levels change over time in individuals at risk or with early symptoms will be crucial for validating its potential as a biomarker. Measurement of different posttranslationally modified forms of α-Syn, such as phosphorylated, nitrated, ubiquitinated, and truncated in CSF may significantly contribute to the diagnosis, monitoring of disease progression, and treatment. Furthermore, combining α-Syn measurements with other biomarkers of neurodegeneration, such as tau or neurofilament light chain, may significantly improve diagnostic accuracy [129].

## Figures and Tables

**Table 1:** Comparison of α-Syn values in PD.

Clinical Assessment	Analyte	Values	Outcome	Ref.
Diagnosis	Total α-Syn	PD vs. controls 1082 pg/mL vs. 1264 pg/mL	Significantly lower in PD compared to controls	[87]
	Total α-Syn	PD vs. controls 1300 pg/mL vs. 1600 pg/mL	Lower in PD compared to controls	[88,89]
	Oligomeric α-Syn S129P Oligomeric/total α-Syn ratio	PD vs. controls 116 pg/mL vs. 57 pg/mL PD vs. controls 261 pg/mL vs. 222 pg/mL PD vs. controls 8.9 vs. 3.5	Significantly higher in PD compared to controls (AUC 0.77, sensitivity 89%, specificity 52%) Higher in PD compared to controls (AUC 0.67, sensitivity 65%, specificity 54%) Significantly higher in PD compared to controls (AUC 0.82, sensitivity 68%, specificity 85%)	[88]
	Oligomeric α-Syn Oligomeric/total α-syn ratio	PD vs. OND Cut-off 2565.5 pg/mL PD vs. OND Cut-off 0.03	Significantly higher in PD compared to OND (AUC 0.72, sensitivity 89%, specificity 48%) higher in PD compared to OND, (AUC 0.78, sensitivity 82%, specificity 64%)	[90]
Differentiation	Total α-Syn	PIGD vs. TD 1185 pg/mL vs. 892.8 pg/mL	Differentiates between TD and PIGD subtype	[87]
	Total α-Syn	PD vs. PDD 204 pg/mL vs. 221 pg/mL	Higher in PDD compared to PD	[91]
	Total α-Syn	PD vs. AD 1372 pg/mL vs. 2912 pg/mL	Differentiates PD from AD	[92]
	Oligomeric α-Syn Oligomeric/total α-Syn ratio	PDD vs. AD 73 309 RLU vs. 26 411 RLU PDD vs. AD 1333 vs. 333	Higher in PDD compared to AD (AUC 0.64) Higher in PDD compared to AD (AUC 0.75)	[93]
Prognosis	Total α-Syn	PD vs. controls at baseline 1763 pg/mL vs. 1975 pg/mL after 2 years 1830 pg/mL vs. 1864 pg/mL	Higher in PD after 2 years compared to controls	[94]
	Oligomeric α-Syn S129P o-α-Syn/t-α-Syn ratio S129P/t-α-Syn ratio	Baseline PD vs. follow-up PD 114.6 pg/mL vs. 181 pg/mL 220.2 pg/mL vs. 180.8 pg/mL 43.4 vs. 48.6 86.5 vs. 47.6	After 2 years a significant increase in oligomeric α-Syn along with o-α-Syn/t-α-Syn ratio, whereas decrease in S129P and S129P/t-α-Syn ratio	[95]
	S129P S129P/total α-Syn ratio	Baseline PD vs. follow up PD 114.66 pg/mL vs. 117.89 pg/mL 0.253 vs. 0.269	Moderate increase after 2 years compared to baseline	[96]
	Total α-Syn	PD vs. controls 68,000 pg/mL vs. 50,500 pg/mL	Higher baseline levels were associated with worsening of cognitive processing speed (AQT) and motor symptoms (UPDRS-III, Hoehn and Yahr, and TUG) over 2-year period	[97]

PD, Parkinson’s disease; AUC, area under the curve; PIGD, postural instability gait difficulty; TD, tremor dominant; RLU, relative light unit; PDD, Parkinson’s disease dementia; AD, Alzheimer’s disease; AQT, a quick test of cognitive speed; UPDRS-III, Unified Parkinson’s Disease Rating Scale-part III; TUG, timed up and go; OND, other neurodegenerative diseases.

**Table 2:** Comparison of α-Syn values in DLB.

Clinical Assessment	Analyte	Values	Outcome	Ref.
Diagnosis	Total α-Syn Oligomeric α-Syn	DLB vs. controls 1400 pg/mL vs. 1800 pg/mL 108 pg/mL vs. 72 pg/mL	Decreased in DLB compared to controls Increased in DLB compared to controls	[101]
	Total α-Syn	DLB vs. controls 161.7 pg/mL vs. 98.2 pg/mL Cut-off 127 pg/mL	Significantly higher in DLB compared to controls AUC 0.79, sensitivity 72%, specificity 85%)	[102]
	Total α-Syn	DLB vs. controls 217 pg/mL vs. 297 pg/mL	AUC 0.72 Combining tau with total α-Syn improved the accuracy of DLB diagnosis compared to α-Syn alone (AUC 0.88)	[91]
	Oligomeric α-Syn/total α-Syn	DLB vs. controls 811 vs. 549	Increased in DLB compared to controls	[93]
Differentiation	Total α-Syn	PD vs. DLB 60.9 pg/mL vs. 161.7 pg/mL Cut-off 128.5 pg/mL	AUC 0.89, sensitivity 72%, specificity 97% Differentiates PD with DLB with good accuracy	[102]
	Total α-Syn	DLB vs. AD 112 pg/mL vs. 183 pg/mL Pro-DLB vs. pro-AD 118 pg/mL vs. 197 pg/mL	Lower in DLB compared to AD AUC 0.75, sensitivity 90.6%, specificity 56.3% AUC 0.83, sensitivity 81.8%, specificity 76.5% Differentiates DLB from AD with moderate diagnostic power; higher α-Syn levels present already at prodromal stages	[100]
	Total α-Syn Total tau/α-Syn	DLB vs. AD 18,100 pg/mL vs. 34,800 pg/mL DLB vs. AD 43 vs. 67	Combination of total tau with α-Syn improves diagnostic accuracy of differentiating DLB from AD	[103]
	Oligomeric α-Syn Oligomeric/total	DLB vs. AD 40.44 RLU vs. 26.44 RLU DLB vs. AD 811 vs. 333	Differentiates DLB from AD with AUC 0.64 Differentiates DLB from AD with AUC 0.75	[93]
	Total α-Syn	DLB vs. AD 111 pg/mL vs. 147 pg/mL	Differentiates DLB from AD, although combined with p-Tau181 increased sensitivity and specificity to 85% and 81%, respectively	[104]
	Total α-Syn	DLB vs. AD 1280 pg/mL vs. 2260 pg/mL Cut-off 1500 pg/mL	Differentiates DLB from AD with AUC 0.85, sensitivity 82%, specificity 76%	[105]
	Total α-Syn	DLB vs. AD 1198 pg/mL vs. 1871 pg/mL Cut-off 1683 pg/mL	Discriminates DLB from AD with AUC 0.82, sensitivity 81.2%, specificity 68.3%	[106]
	Total α-Syn	DLB vs. AD 1751.1 pg/mL vs. 2450.8 pg/mL	Differentiates DLB from AD	[107]

DLB, dementia with Lewy bodies; PD, Parkinson’s disease; AD, Alzheimer’s disease; AUC, area under the curve; pro-DLB, prodromal DLB; pro-AD, prodromal AD; RLU, relative light units; p-Tau181, tau phosphorylated at serine 181.

**Table 3:** Comparison of α-Syn values in MSA.

Clinical Assessment	Analyte	Values	Outcome	Ref.
Diagnosis	Total α-Syn	MSA vs. controls 1110 pg/mL vs. 2220 pg/mL	Significantly lower in MSA compared to controls	[112]
	Total α-Syn	MSA vs. controls 300 pg/mL vs. 480 pg/mL Cut-off 460 pg/mL	Significantly lower in MSA compared to controls AUC 0.88, sensitivity 94%, specificity 70%	[113]
	Total α-Syn	MSA vs. controls 750 pg/mL vs. 1310 pg/mL	Significantly lower in MSA compared to controls	[114]
	Total α-Syn	MSA vs. controls 108 pg/mL vs. 137.8 pg/mL		[115]
	Total α-Syn	MSA vs. controls 1347 pg/mL vs. 1782 pg/mL		[116]
Differentiation	Total α-Syn	MSA vs. PD 1110 pg/mL vs. 1340 pg/mL	Differentiates MSA from PD	[112]
	Total α-Syn	MSA vs. AD 300 pg/mL vs. 550 pg/mL cut-off 320 pg/mL	Differentiates MSA from AD with AUC 0.92, sensitivity 95%, specificity 70%	[113]
	Total α-Syn	MSA vs. PD 750 pg/mL vs. 840 pg/mL	Differentiates MSA from PD	[114]
	Total α-Syn	MSA vs. AD 108 pg/mL vs. 184.1 pg/mL		[115]
	Total α-Syn	MSA vs. PD 1347 pg/mL vs. 1767 pg/mL		[116]
	Oligomeric α-Syn S129P Oligomeric S129P	MSA vs. PD 22,490,000 pg/mL vs. 7,040,000 pg/mL MSA vs. DLB 22,490,000 pg/mL vs. 9,470,000 pg/mL MSA vs. PD 7,140,000 pg/mL vs. 3,430,000 pg/mL MSA vs. DLB 7,140,000 pg/mL vs. 1,630,000 pg/mL MSA vs. PD 19,560,000 pg/mL vs. 770,000 pg/mL MSA vs. DLB 19,560,000 pg/mL vs. 1,600,000 pg/mL	Significantly higher in MSA compared to PD Higher in MSA compared to DLB Significantly higher in MSA compared to PD Significantly higher in MSA compared to DLB Significantly higher in MSA compared to PD Significantly higher in MSA compared to DLB Both oligomeric and phosphorylated α-Syn differentiate MSA from PD and DLB	[117]
	S129P	MSA vs. AD 58.12 pg/mL vs. 72.64 pg/mL	Differentiates MSA from AD	[118]

MSA, multiple system atrophy; AUC, area under the curve; PD, Parkinson’s disease; AD, Alzheimer’s disease, DLB, dementia with Lewy bodies; S129P, α-synuclein phosphorylated at serine 129.
